# Tailoring Performance, Damping, and Surface Properties of Magnetorheological Elastomers via Particle-Grafting Technology

**DOI:** 10.3390/polym10121411

**Published:** 2018-12-19

**Authors:** Martin Cvek, Miroslav Mrlik, Jakub Sevcik, Michal Sedlacik

**Affiliations:** Centre of Polymer Systems, University Institute, Tomas Bata University in Zlín, Trida T. Bati 5678, 76001 Zlín, Czech Republic; cvek@utb.cz (M.C.); mrlik@utb.cz (M.M.); j4sevcik@utb.cz (J.S.)

**Keywords:** magnetorheology, silicone elastomer, polymer grafting, damping, ATRP, surface properties

## Abstract

A novel concept based on advanced particle-grafting technology to tailor performance, damping, and surface properties of the magnetorheological elastomers (MREs) is introduced. In this work, the carbonyl iron (CI) particles grafted with poly(trimethylsilyloxyethyl methacrylate) (PHEMATMS) of two different molecular weights were prepared via surface-initiated atom transfer radical polymerization and the relations between the PHEMATMS chain lengths and the MREs properties were investigated. The results show that the magnetorheological performance and damping capability were remarkably influenced by different interaction between polydimethylsiloxane chains as a matrix and PHEMATMS grafts due to their different length. The MRE containing CI grafted with PHEMATMS of higher molecular weight exhibited a greater plasticizing effect and hence both a higher relative magnetorheological effect and enhanced damping capability were observed. Besides bulk MRE properties, the PHEMATMS modifications influenced also field-induced surface activity of the MRE sheets, which manifested as notable changes in surface roughness.

## 1. Introduction

In recent years, magnetorheological elastomers (MREs) have attracted much attention in scientific as well as industrial fields due to their rapidly-tunable viscoelastic properties once they are exposed to an external magnetic field. The MREs represent composite systems comprising the ferromagnetic particles embedded in an elastomeric matrix thus they can be perceived as solid analogues to the magnetorheological fluids (MRFs) [1]. The indisputable advantage of the MRFs over the MREs is the ability to increase their rheological properties (yield stress, viscosity, viscoelastic moduli) by several orders of magnitude [2,3]. In the MREs, the magneto-induced effects are generally less pronounced however, their application can eliminate a serious sedimentation stability problem of the MRFs [3,4,5,6]. Moreover, other common drawbacks of the MRFs such as sealing issues, possible leakage or environmental contamination can be successfully suppressed [5]. High stability and tunable viscoelastic moduli changes make the MREs suitable materials for the applications such as vibration absorbers, adaptive dampers, or stiffness tunable mounts [7,8,9,10]. Undoubtedly, pioneer applications such as artificial muscles [11], micro-fluid transport systems [12], radio-absorbers [13], sensors [14], or active elements of electric circuits [15] based on the MREs concept have been reported.

Magnetic, rheological, and mechanical properties of the MREs can be affected by many variables during their fabrication process; among the most important aspects belong the particle concentration, their size, magnetic properties, spatial distribution within the matrix, etc. These phenomena were recently studied by Khimi et al. [16] who due to complexity of the issue designed the Taguchi method (a statistical method identifying the performance trends among multiple factors and determining their combination that yields the optimum results) to investigate the effects of a number of factors (particle concentration, size and distribution, applied magnetic field during curing). They found that the particle concentration had the greatest influence on studied quantity, namely, the loss tangent, followed by the effect of particle size and intensity of applied field. 

Besides the particle-related factors, the selection of matrix also plays a key role in designing the effective MREs. Generally, the matrix should be highly elastic to retain its shape [17], while allowing the rearrangement of the particles to enhance the damping properties at the same time [18]. In some applications, the weight and flexibility of the matrix are desired thus the MREs based on porous matrices can be preferred [19]. Recently, Fan et al. [20] showed that the notable increase in the MRE damping properties can be evoked by a reduction of the binding force of the matrix exerted on the particles. In their study, such reduction was a consequence of reduced cross-link density of the *cis*-polybutadiene rubber (BR) matrix. Other approach in controlling the MREs damping properties involves the addition of polycaprolactone (PCL) as a temperature-controlling component also into the BR matrix. The PCL can transform from a semicrystalline solid to a liquidated soft material once the surrounding temperature is increased above the PCL melting point [21]. The damping properties of such MREs can be controlled by varying the PCL content and a temperature. Also, the addition of non-phthalate ester plasticizer (sucrose acetate isobutyrate) into the epoxidized natural rubber was found to soften the matrix and improve relative MR effect of as-modified MREs [22]. Rabindranath et al. [23] found that final MREs’ properties and stress transfer between the particles and the matrix can be efficiently modulated by an incorporation of the particles treated with various surfactants such as fatty acids or calcium and aluminum soaps. Their MRE was, however based on the polydimethylsiloxane (PDMS). It has to be mentioned that bare carbonyl iron (CI) particles were less mobile in such matrix due to the possible covalent bonding between the hydroxyl groups on their surface and the silane groups of the cross-link agent. Unfortunately, introducing the surfactants into the MREs can initiate several issues related to the durability of these systems. The surfactants are not covalently bonded onto the magnetic substrate, thus their diffusion through the polymer matrix can arise. In addition, the presence of common plasticizers can cause durability problems as these low-molecular weight substances have a tendency to migrate through the polymer matrix [24]. Although the reviewed approaches are effective in tuning the MREs properties, they rather omit a protection of the incorporated particles against high temperatures or acidic environment, which is essential in some practical applications [25]. Thus, there is a need to address the demands related to both performance and stability properties of the MREs, ideally in a single-step way.

In our previous study [26], we have shown that the CI particles covalently grafted with poly(trimethylsilyloxyethyl methacrylate) (PHEMATMS) chains (CI-*g*-PHEMATMS) via surface-initiated atom transfer radical polymerization (SI-ATRP) exhibited high thermo-oxidation stability, excellent chemical stability, enhanced dispersibility and significant wettability with PDMS matrix. Moreover, the presence of the PHEMATMS grafts restricted the covalent bonding between the CI particles and the PDMS, which resulted in increased relative MR effect and damping factor.

Herein, we present a novel method to tailor the MR performance, damping properties, and its impact on the surface activity of the MREs. The core of this method stems from the incorporation of the particles grafted with PHEMATMS of different molecular weights, which are expected to affect the PDMS cross-link density to a various degree, which ultimately can lead to different particle rearrangements and MREs performance. The SI-ATRP was chosen as a suitable technique as the thickness of grafted polymer layer is a crucial parameter to preserve sufficient magnetization of the particles [27] and it can be easily controlled using this polymerization technique. The goal of this study was to investigate the effect of different polymer graft lengths on the above-described characteristics, and thus to be able to modify bulk as well as surface behavior of the MREs through controllable coating of the magnetic particles, which is to the best of our knowledge a concept that has never been published elsewhere before.

## 2. Materials and Methods 

The CI particles (SL grade) supplied by BASF Corporation (BASF, Ludwigshafen, Germany) represented a suitable magnetic substrate. The 2-hydroxyethyl methacrylate (HEMA, 96%) and chlorotrimethyl silane (TMCS, ≥97%) were used as the components to prepare trimethylsilyloxyethyl methacrylate (HEMATMS) monomer in dichloromethane (anhydrous, ≥99.8%) solution, while triethylamine (TEA, ≥99%) served as a proton scavenger. The (3-aminopropyl)triethoxysilane (ATPES, ≥98%) was utilized as a linker to further bond the *α*-bromoisobutyryl bromide (B*i*BB, 98%) initiator attached to the particle surface. The ethyl α-bromoisobutyrate (EB*i*B, 98%) was used as a sacrificial initiator, while the *N,N,N′,N″,N″*-pentamethyldiethylenetriamine (PMDETA, ≥99%) served as a ligand increasing the solubility of copper bromide (CuBr, ≥99%) initiator. The ATRP was performed in the anisole (99%). Aluminum oxide (neutral) is a depriving agent employed to remove the catalyst before the chromatography measurements. All chemicals were purchased from Sigma-Aldrich (St. Louis, MO, USA). Solvents and purification agents, namely tetrahydrofurane (THF, p.a.), acetone (p.a.), ethanol (absolute anhydrous, p.a.), toluene (p.a.), and hydrochloric acid (HCl, 35%, p.a.) were obtained from Penta (Penta, Prague, CZ) and used as received. The MREs were prepared using Sylgard 184/catalyst kit (Dow Corning, Atlanta, GA, USA).

### 2.1. Monomer Synthesis and Preparation of CI-g-PHEMATMS Particles

**Figure 1:**
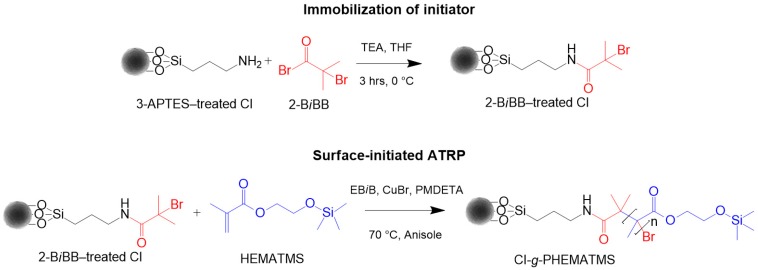
Preparation scheme of the CI-*g*-PHEMATMS via SI-ATRP.

The HEMATMS was synthesized via esterification reaction of argon-purged HEMA and freshly distilled TMCS while the CI-*g*-PHEMATMS particles were prepared following the procedures reported previously [26]. The preparation scheme of grafting the CI particles via SI-ATRP is outlined in Figure 1. To obtain the particles varying in shell thickness, and different monomer-to-initiator ratios were employed (Table 1, sample code 1 and 2). While in the first-case synthesis the same material was obtained as previously [26], herein we further designed the second type of the particles, which gave us the opportunity to study the effect of PHEMATMS molecular weight on bulk as well as surface MREs’ properties. The both variants of grafted CI particles were thoroughly washed in THF, ethanol, and acetone following the procedures [27] and were further used for the fabrication of the MREs.

### 2.2. Fabrication of MREs

The isotropic MREs containing bare CI particles or two variants of their PHEMATMS-grafted analogues were fabricated. The particles fraction in the MREs were maintained at 60 wt %. Firstly, the silicone elastomer and its curing agent (weight ratio of 20:1) were thoroughly mixed for 10 minutes; subsequently the specified amount of magnetic particles was added followed by mechanical mixing using a pestle for additional 10 min at laboratory temperature to obtain well-dispersed systems. The resulting mixtures were degassed for 20 min under 10 mbar and finally poured into the PTFE molds. The curing was performed in an oven pre-heated to 100 °C for 45 min. The corresponding products were thin MRE sheets, from which the specimens were cut out. 

### 2.3. Characterization of PHEMATMS Coatings

Purity of monomer and polymerization progresses during SI-ATRP were investigated using nuclear magnetic resonance spectroscopy (1H NMR, 400 MHz VNMRS Varian, Tokyo, Japan) equipped with 5 mm 1H-19F/15N-31P PFG AutoX DB NB probe at 25 °C using deuterated chloroform as a solvent. Apparent molecular weights (weight, ${\bar{M}}_{W}$, and number, ${\bar{M}}_{n}$, averages) and dispersity indexes (*Đ*) were determined using gel permeation chromatography (GPC, PL-GPC220, Agilent, Hachioji, Japan) consisted of a Waters 515 pump, two PPS SDV 5 µm columns (*d* = 8 mm, *l* = 300 mm; 500 Å + 105 Å) and a Waters 410 differential refractive index detector with THF as an eluent at flow rate of 1.0 mL·min^−1^. The instrument was calibrated using polystyrene standards, while anisole served as an internal standard to correct possible fluctuations in THF flow rate.

### 2.4. Characterization of Core-Shell Particles

Fourier-transform infrared spectroscopy (FTIR) was utilized in attenuated total reflectance mode to prove the presence of PHEMATMS layer grafted onto the CI surface. The FTIR examination was conducted on Nicolet 6700 (Thermo Scientific, Waltham, MA, USA) under laboratory conditions employing the Ge crystal in a typical wavenumber region of 3600–600 cm^−1^. To observe the thicknesses of grafted layers, the transmission electron microscopy (TEM) images were acquired using JEM-2100Plus (Jeol, Tokyo, Japan). TEM samples were prepared by ultrasonic dispersion (Sonopuls HD 2070, Bandelin electronic, Berlin, Germany) of the particles in acetone and dropping them onto a carbon coated copper grid (300 mesh, Agar Scientific, Standsted, UK). In order to limit the particle drifts during the image acquiring, the second copper grid was used as a protection cover. The effects of grafted PHEMATMS layers on particle density and magnetic properties were analyzed using a gas pycnometry (UltraFoam 1200e, Quantachrome Instruments, Odelzhausen, Germany) with nitrogen as a gaseous medium, and a vibrating-sample magnetometry (VSM, Model 7404, Lake Shore, Westerville, OH, USA) in a range of ±10 kOe (±780 kA·m^−1^), respectively. The VSM measurements were performed on precisely-weighted raw powders, and their corresponding MREs accommodated in the VSM sample holder (730931 Kel-F, powder/bulk upper/bottom cup). Both analyses were performed at ambient temperature. The presented density data are the average values of the measurements from five samples with five measurements for each sample, which is a similar procedure as published elsewhere [28]. The thermogravimetric analysis (TGA) of the particles was investigated with the help of a TGA device (TA Instruments, Q500, New Castle, DE, USA) using nitrogen as a purge gas with a flow rate of 60 mL·min^−1^. For each test, the sample mass of approx. 10 mg was exposed to temperature range of 35–600 °C, while the heating rate was set to 5 °C·min^−1^. The chemical stability of the particles was examined by a facile corrosion test, in which 1 g of particles were dispersed in 20 mL of 0.1 M aqueous HCl acid solution at laboratory temperature and the pH-value was recorded as a function of time with the help of calibrated pH meter (SensoDirect pH110, Tintometer, Dortmund, Germany). The conditions (0.1M HCl) for this test were specifically chosen to suitably accelerate the corrosion process of tested materials.

### 2.5. Characterization of MREs

The microstructure of the samples was observed by using the scanning electron microscopy (SEM, Phenom Pro, Phenom-World, Eindhoven, The Netherlands). The presented images were taken on the freeze-fractured MRE surfaces at an accelerating voltage of 5 kV.

Magnetorheological performance of the MREs was studied using a rotational rheometer Physica MCR502 (Anton Paar, Graz, Austria) equipped with a magneto-device (Physica MRD 180/1T) and the parallel-plate geometry (PP20/MRD/TI) with a diameter of 20 mm. The circular MRE specimens of thickness approximately 1.15 mm were subjected to external magnetic field (0–860 kA·m^−1^ corresponding to coil current of 0–3 A), which was generated by a power source (PS-MRD/5A) and their proper contact with the geometry was ensured by applying an initial normal force of 0.85 N. All the viscoelastic measurements were carried out at a constant temperature of 25 °C, which was maintained by a temperature control unit (Julabo FS18, Seelbach, Germany). The amplitude sweeps were performed employing a range of deformation amplitude from 10^−3^ to 10% and a constant frequency of 5 Hz. Subsequently, the storage, *G′*, and the loss, *G″*, moduli as well as the damping factor, tan *δ*, of the MREs were collected in the magneto sweep measurements, which were performed in ascending/descending magnetic fields at a frequency of 5 Hz and a constant amplitude strain of 0.2% (in all cases the linearity of the sample response was verified). As known, after several magneto-shear-loading cycles the viscoelastic moduli saturates and the stable values can be obtained [29]. After such pre-conditioning, the frequency sweeps were performed in a range from 10^−1^ to 4 × 10 Hz.

The optical profilometry was performed on small circular specimens (diameter of 4.5 mm), which were cut out from 0.35 mm thin MRE sheets. The surface roughness of the specimens was investigated in the absence as well as in the presence of the external magnetic field, which was generated using a cylindrical NdFeB magnet (height of 1 mm, diameter of 5 mm, magnetic flux density of ≥350 mT). The effect of magnetic field on the surface roughness of the MRE sheets was studied with the help of the Contour GT-K 3D optical profilometer (Bruker, Leipzig, Germany) equipped with the parfocal interferometric objective (magnification of 2.5×) with zoom lens (magnification of 1×). The topological changes occurring after the magnetic field exposure were qualitatively assessed using vertical scanning interferometry method. The three surface areas of 1 × 1 mm^2^ were analyzed in detail on each sample. The presented results are the average values from three independent measurements.

## 3. Results

### 3.1. Synthesis

The synthesis of CI-*g*-PHEMATMS particles using SI-ATRP involves the end-linking of HEMATMS monomer units onto B*i*BB-treated particles, having homolytically cleavable alkyl-bromide bond, via radical coupling reaction. Generally, in ATRP the polymer chain lengths can be controlled by monomer-to-initiator ratio in combination with monomer conversion [30], which can be affected by temperature, reaction time, type of transition metal and ligand used, solvent, etc. Herein, the reactions were conducted under various stoichiometry when the monomer-to-initiator molar ratio was altered (Table 1, sample code 1 and 2). In order to obtain better control over the molar ratio and enable determination of molecular characteristics of the polymer, a sacrificial initiator was used in excess when compared to initiator bonded on the CI surface. As a result, well-defined CI-*g*-PHEMATMS particles of two different molecular weights with narrow *Đ* and thus different shell thicknesses were obtained. The monomer conversions determined from ^1^H NMR spectra and molecular characteristics from GPC are listed in Table 2.

The successful modification of the CI particles with PHEMATMS polymer grafts was proved via FTIR where peaks indicating the characteristic groups were revealed [26]. In the FTIR spectra of CI-*g*-PHEMATMS, the –CH stretching from –CH_3_ and –CH_2_– groups was observed at around 3000 cm^−1^. Furthermore, the peak at 1720 cm^−1^ was attributed to C=O stretching originated from pendant groups of B*i*BB and HEMATMS constituents. Raised absorption levels at 1082 and 1041 cm^−1^ were distinct indications of organosilicon moieties, specifically resulting from Si–O–Si stretching. In addition, the peak occurring at 849 cm^−1^ was assigned to be –Si–CH_3_ rocking that confirmed silyl-based nature of the organic shell. These wavenumber values were found to be in a good agreement with the literature [31].

### 3.2. Thicknesses of Grafted Layers

**Figure 2:**
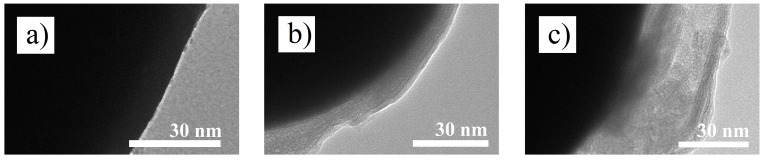
TEM images of (**a**) bare CI, (**b**) CI-*g*-PHEMATMS-1, and (**c**) CI-*g*-PHEMATMS-2 showing a part of the corresponding single particle.

The TEM images (Figure 2) clearly show the presence of a lower-contrast shell grafted onto the darker CI core, thus the desired core-shell structure has been successfully revealed. As seen, the grafted layers were generally uniform in both cases of modified particles as a result of effective SI-ATRP. Due to PHEMATMS modification the density of the particles decreased from ~7.79 to ~7.35 and ~7.12 g·cm^−3^, respectively, which is in accordance with the observed thicknesses of layers being ~15 nm and ~35 nm for CI-*g*-PHEMATMS-1 and CI-*g*-PHEMATMS-2, respectively. The determined thickness values are slightly higher than those calculated for fully-stretched polymer chains based on molecular weights of PHEMATMS (11 and 22 nm, respectively), most probably due to different hydrodynamic volume of PHEMATMS in comparison with polystyrene, which was used as a standard in the GPC system. Nevertheless, the results are in a good agreement with the expectation that the polymer layer thickness increases with the increasing molecular weight of the grafted polymer.

### 3.3. Magnetic Properties

**Figure 3:**
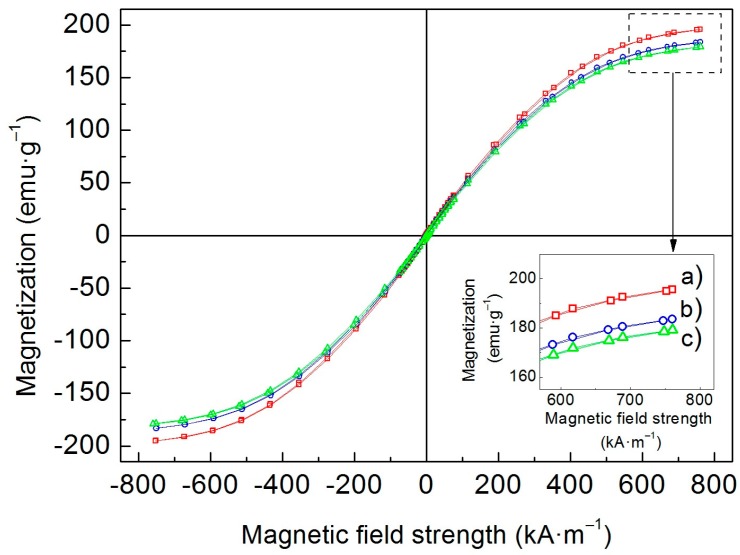
Magnetization curves of (**a**) bare CI, (**b**) CI-*g*-PHEMATMS-1, and (**c**) CI-*g*-PHEMATMS-2 particles obtained via VSM. The inset figure shows the data differences near the saturation magnetization.

As known, particle magnetization is a crucial factor to obtain high MR effect [3]. Figure 3 represents the magnetization curves of bare CI particles and both of their grafted analogues. Having grafted non-magnetic material, the mass magnetization of resulting core-shell structures decreases. However, the decrease in magnetization was relatively small for CI-*g*-PHEMATMS-1 and slightly more pronounced for CI-*g*-PHEMATMS-2 particles as the thickness of polymer layer was in nanometric scale (Figure 2). These results are in agreement with the recent data obtained on poly(glycidyl methacrylate)-modified CI particles, which were successfully used to prepare stable MR suspensions [32]. It is also important to mention, that the observed magnetization decrease was minimal when compared to similar core-shell structures, such as the CI particles grafted with polydopamine via an oxidative self-polymerization. Dopamine coating attenuated particle magnetization roughly by 36% near saturation at comparable fields as shown recently [33]. The results indicate that SI-ATRP is a suitable tool for controllable modification of magnetic substrate.

Furthermore, the magnetization of the MREs was investigated. As clarified by de Vicente et al. [34], motion and orientation of magnetic fillers can notably influence magnetic response of the composite samples. In this sense, Stepanov et al. [35] performed susceptibility measurements using the MREs of different stiffness (Young’s modulus of 60, and 400 kPa, respectively) revealing that softer MREs are characterized by more pronounced magnetic hysteresis. It is supposed that in softer MRE, the field-induced motion of the particles locally deforms the matrix, which creates new relatively-stable state since negative gain in magnetic energy is much higher than the increment of the elastic one [35]. In another study [36], the temperature dependence of this phenomenon was investigated. It was found that magnetic hysteresis of the same MRE was rather significant at high temperature (310 K), and non-existent at lower one (170 K). This behavior is again strongly related to temperature dependence of polymer matrix stiffness and mobility of the embedded particles.

**Figure 4:**
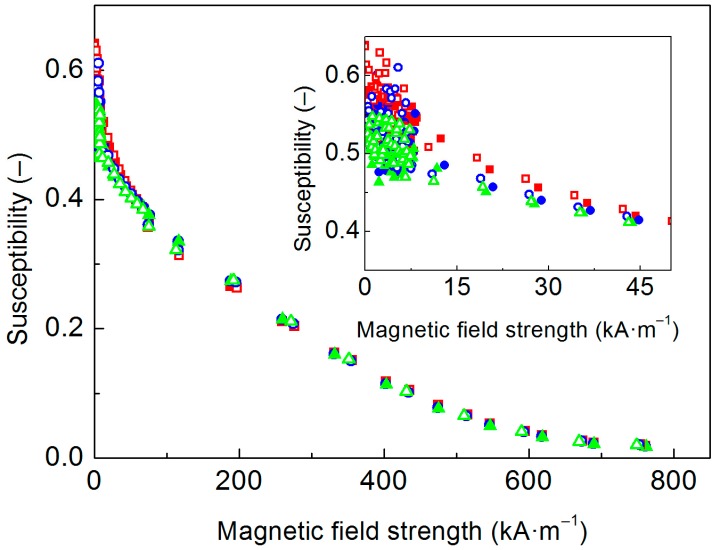
Differential susceptibility curves of the MREs containing (**red squares**) bare CI, (**blue circles**) CI-*g*-PHEMATMS-1, and (**green triangles**) CI-*g*-PHEMATMS-2 particles. The closed/open symbols denote data for decreasing/increasing magnetic field, while the inset magnifies the low-field susceptibilities.

As will be shown in Section 3.5, in our case the matrix stiffness was modulated by different particle/matrix interactions due to performed ATRP, therefore magnetic hysteresis investigation was of our interest. Figure 4 illustrates the susceptibility of the investigated MREs. As can be seen, low-field susceptibilities of the MREs containing the CI-*g*-PHEMATMS particles were slightly lower when compared to the reference. This feature can be explained as a consequence of decreased magnetization due to non-magnetic PHEMATMS layers (Figure 3). At higher fields, however, the susceptibilities of all three MREs were comparable. It can be asserted that PHEMATMS-grafted particles exhibited higher relative motions within the PDMS when compared to bare CI particles, and as a result they grouped into the chains enhancing the MRE susceptibility, that approached to the susceptibility of the MRE containing bare CI particles. The data are consistent also with mechanical characteristics of the MREs (shown later). 

Nevertheless, none of the tested MREs exhibited an appreciable magnetic hysteresis. This fact can be explained on the basis of small differences in stiffness among the samples. In the previous papers, the matrix stiffness differed by almost the magnitude [35] or it was caused by severe temperature change [36]. The observed data thus mostly collapse with the hysteresis-free situation observed by Stepanov et al. [35] on their stiffer MRE variant.

### 3.4. Stability Properties of CI-g-PHEMATMS

The MREs can be exposed to demanding operating conditions such as high temperatures or acidic reactive species (e.g., acid rains, sea humidity, operating fluid leakage), which are factors affecting their long-term stability and durability properties [4]. In our previous paper, we have shown that the presence of compact PHEMATMS layer prevented the oxidation of the CI core, and remarkably shifted the thermo-oxidation processes to higher temperatures. 

**Figure 5:**
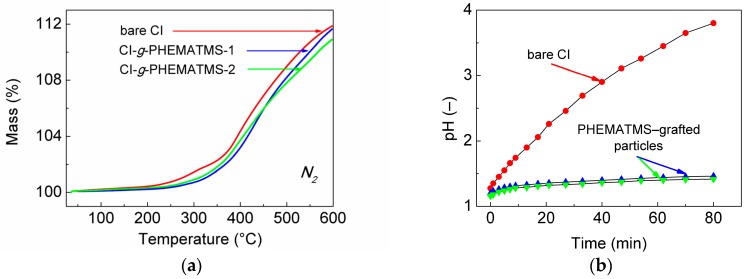
Representations of (**a**) TGA curves, and (**b**) resistance to acidic conditions of bare CI particles, and their PHEMATMS-grafted analogues.

For the short-time exposures of the MREs to high temperatures, the diffusion of the oxygen can be neglected. Therefore, we present the TGA of the particles analyzed in the inert atmosphere. As seen in Figure 5a, the mass increase with temperature was observed for all the studied particles. Generally, the TGA curves were characterized by stability period up to 200 °C followed by a region around 300 °C showing a slight enhancement in thermal stability for the PHEMATMS-grafted particles when compared to the reference. Finally, the gradual mass increase was observed for all particle types reaching the mass increment of around 11% at the temperature around 600 °C. Similar phenomenon was reported also by Abshinova et al. [37], but the explanation was not given. We assume that this increase can be the most probably explained as a consequence of the nitridic phase development, which is typical for the CI particles [38]. Figure 5b further shows the results from an anti-acid/corrosion test. As known, bare CI particles are relatively unstable in acidic environment, which leads to a formation of less magnetic products [39,40]. On the contrary, both variants of CI-*g*-PHEMATMS particles were extremely stable under these acidic conditions, regardless of the thickness of the layer, which imply that the PHEMATMS layers were uniform without any defects and the iron cores were efficiently protected. The result suggests that also the performance of modified MREs could be sufficiently preserved after their acid treatment similarly as reported earlier on the systems with embedded tetraethoxysilane–coated CI particles [4].

### 3.5. Magnetorheological Performance

**Figure 6:**
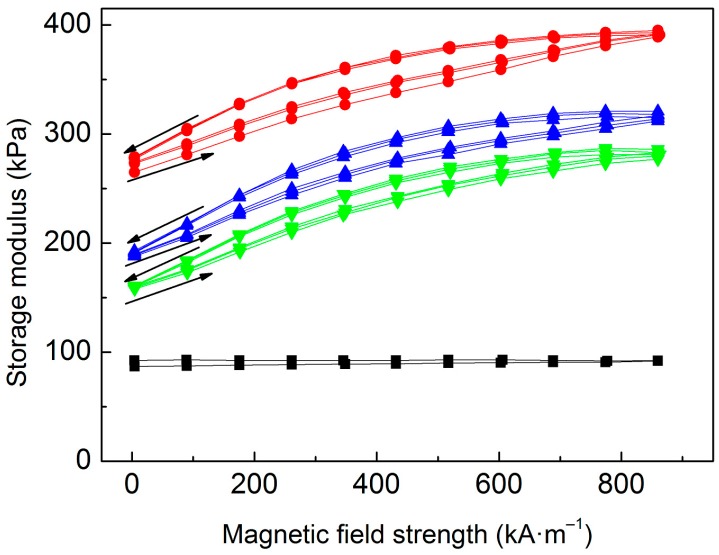
Storage modulus of neat PDMS matrix (**black squares**), and the MREs containing bare CI (**red circles**), CI-*g*-PHEMATMS-1 (**blue up-triangles**), and CI-*g*-PHEMATMS-2 (**green down-triangles**) particles as a function of applied magnetic field strength.

**Figure 7:**
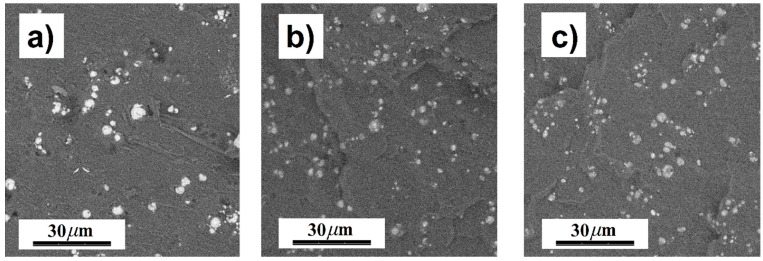
SEM images of the MREs containing (**a**) bare CI, (**b**) CI-*g*-PHEMATMS-1, and (**c**) CI-*g*-PHEMATMS-2 particles.

To understand the effect of PHEMATMS molecular weight on the MR behavior, the MREs were subjected to oscillatory shearing under an external magnetic field with ascending/descending character due to their hysteresis behavior [29]. Figure 6 displays the *G′* dependence on applied magnetic field strength, *H*. Obviously, the lowest *G′* exhibited neat PDMS matrix, while in the final MREs the *G′* was readily increased by adding the micro-particles since the rigid inorganic matter have a much higher stiffness than the polymer matrix [41]. The highest *G′* possessed the MRE containing bare CI particles due to already mentioned particle/matrix covalent bonding between the hydroxyl groups on the metal surface and the silane groups of PDMS curing agent [23]. Despite bonding phenomenon certain particle/matrix incompatibility can be presumed due to different polarity [26] of bare CI and PDMS (Figure 7). The interphase incompatibility in the MRE containing bare CI will be further discussed in a connection to damping capabilities.

In the MREs containing CI-*g*-PHEMATMS particles the covalent bonding was restricted, thus the particle/matrix interactions were executed by the physical entanglements between the PHEMATMS grafts and PDMS chains. As known, the interactions provided via polymer chain entanglements are weaker than those provided via covalent bonds, which resulted in lowering the *G′* of the MREs containing the PHEMATMS-grafted magnetic particles (Figure 6). When the molecular weight of PHEMATMS was increased, the *G′* of modified MRE further decreased. This trend can be attributed to the different level of polymer chain entanglement in the vicinity of modified particles. It appears that the presence of ATRP–grafted PHEMATMS can increase the mobility of CI particles, and this effect is dependent on the PHEMATMS molecular weight. Similar results were drawn by Fan et al. [18] who observed an analogous phenomenon by adding a different amounts of naphthenic oil as a plasticizer into the BR matrix. The results not only suggest a plasticizing effect of the PHEMATMS grafts, but also describe the influence of their molecular weight. The opposite behavior was observed recently by Gorodov et al. [42] who modified the CI particles by carboxyl-containing PDMS and embedded these into the PDMS matrix. Due to strong CI-PDMS/PDMS interactions they observed rather stiffening response, and thus lowered MR effect in their modified MREs. The results indicate that grafting technology and the combination of materials play a significant role in the final behavior of the MRE composite.

**Figure 8:**
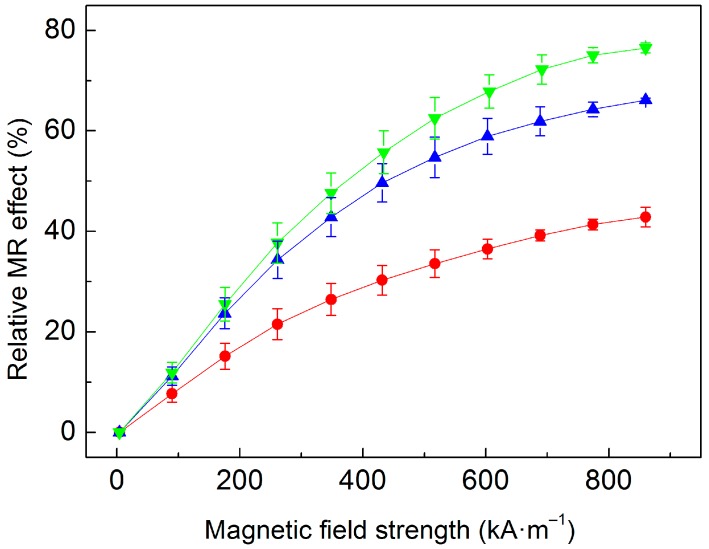
Relative MR effect of the MREs containing bare CI (**red circles**), CI-*g*-PHEMATMS-1 (**blue up-triangles**), and CI-*g*-PHEMATMS-2 (**green down-triangles**) particles as a function of applied magnetic field strength.

To further evaluate the plasticizing effect of our PHEMATMS-grafted systems, relative MR effect, *e*, formalism was applied. This quantity can be calculated according to the Equation

(1) $$e=\left(G_{H}^{\prime }-G_{0}^{\prime }\right)\;/\;G_{0}^{\prime }\times 100$$

where $G_{H}^{\prime }$ is the field-on *G′* (at a certain magnetic field) and $G_{0}^{\prime }$ is the field-off *G′*. As seen in Figure 8, the *e* of studied MREs showed the increasing trends with increasing *H* almost reaching the saturation plateau at high *H*. The employed magneto-cell is capable of reaching even higher magnetic fields, however those were not used due to possible inhomogeneity of magnetic field profile as demonstrated by Laun et al. [43] Based on the results observed in Figure 8, it is obvious, that the MRE containing bare CI particles exhibited the lowest relative MR effect, while its analogues containing PHEMATMS-grafted particles exhibited higher ones. This phenomenon was the most significant in the MRE containing the CI-*g*-PHEMATMS-2 particles probably due to loosen cross-link density caused by higher molecular weight grafts, which facilitated the particle rearrangement. Therefore, despite slightly lower mass magnetization of modified particles (Figure 3), their MREs were able to develop higher relative MR effects. A non-linear relation between the PHEMATMS molecular weight and the maximum MR effect was found. By increasing the molecular weight from 9200 to 17,900 g·mol^−1^ the maximum (at 860 kA·m^−1^) relative MR effect increased by a factor of 1.54 and 1.78, respectively, when compared to the reference. This result suggests that the theoretical maximum of PHEMATMS molecular weight exists, above which the MR effect no longer increases. 

**Figure 9:**
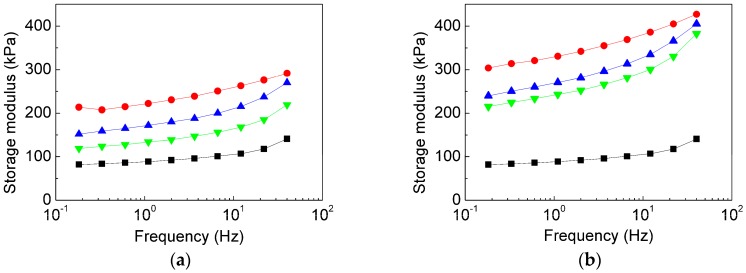
(**a**) Off-state and (**b**) the on-state (860 kA·m^−1^) storage modulus, *G′*, of neat matrix (**black squares**), and the MREs containing bare CI (**red circles**), CI-*g*-PHEMATMS-1 (**blue up-triangles**), and CI-*g*-PHEMATMS-2 (**green down-triangles**) particles as a function of frequency.

Next, the effect of frequency on the *G′* for the MREs containing bare CI or their PHEMAMT-grafted analogues was studied. The off-state (Figure 9a) as well as the on-state (Figure 9b) data in terms of the *G′* values were in accordance with those obtained from the magneto-sweep experiments (Figure 6). As seen, the *G′* of all the samples was sensitive to the excitation frequency, which is a typical behavior for a viscoelastic material [44]. At higher frequencies (a shorter timescale), the elastic portion of their complex behavior is usually resulting in higher *G′*; and this tendency was obvious in all our studied samples (Figure 9). The frequency dependency was also clearly apparent for neat matrix as the relaxation characteristics of PDMS molecular chains were not disturbed [44]. The inclusion of bare CI particles obstructed the motion of PDMS molecules due to already mentioned particle/matrix covalent bonding leading to a reduction of frequency dependence phenomenon. On the contrary, the presence of both variants of PHEMATMS-grafted CI particles reduced the cross-link density supporting the movement of the matrix molecular chains, which resulted in a steeper *G′* increase with frequency especially above 10 Hz.

**Figure 10:**
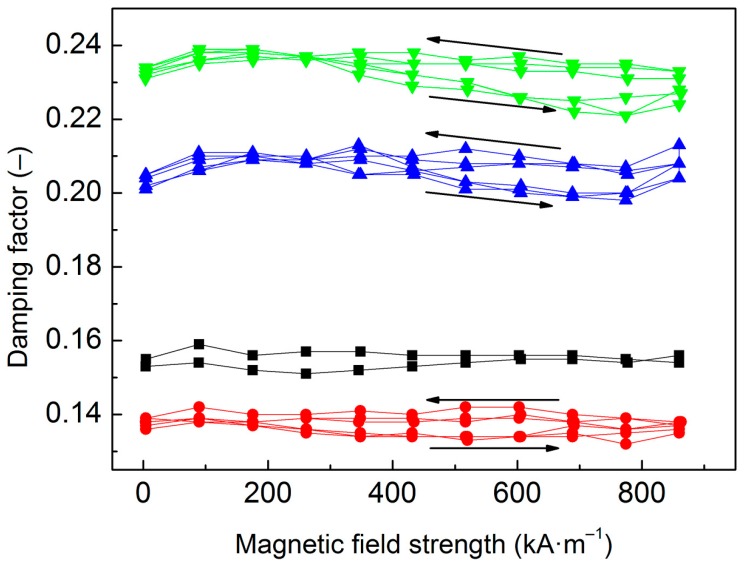
Damping factor, tan *δ*, of neat PDMS matrix (**black squares**), and the MREs containing bare CI (**red circles**), CI-*g*-PHEMATMS-1 (**blue up-triangles**), and CI-*g*-PHEMATMS-2 (**green down-triangles**) particles as a function of applied magnetic field strength.

Furthermore, the effect of the PHEMATMS molecular weight on the damping capacity of the MREs was investigated (Figure 10). Based on the literature, the topic of the damping of the MREs can still evoke a controversy—while in some papers it is stated that magnetic field does not have any remarkable effect on damping [45], other scientists claim that damping of the MREs changes due to the applied magnetic field [5]. Recently, Shuib et al. [46] proposed a mathematical damping model, which matched well with the experimental trends showing that the damping capacity of the MREs is dependent on magnetic field up to magnetic saturation. Herein, the employed magnetic fields were still below the particle magnetic saturation [32], thus the total damping is further discussed as the average value of tan *δ* obtained through the whole *H* range. The total damping capacity of the MREs includes several mechanisms, namely damping by the viscous flow of the elastomeric matrix, interfacial damping, and magnetism-induced damping [46].

The first-mentioned mechanism is provided by the viscoelastic polymer matrix, which is typically characterized following the Kelvin-Voight model [46]. In the interfacial damping, two classes are distinguished. The damping in the systems with strongly bonded interfaces (matrix molecules are bonded to the surface of the filler particles) can be attributed to the energy required to bring about viscous flow of constrained materials in the vicinity of the particle interfaces [46,47]. The damping in the systems with weakly bonded interfaces is caused mainly due to internal friction between the particles and matrix and can be estimated based on Lavernia’s analysis [48]. Recently, it was found that this damping mechanism can be significantly improved by the incorporation of the functionalized carbon nanotubes (1 wt %) into the body of the MREs [49]. Finally, magnetism-induced damping is represented by the energy absorbed to overcome magnetic interactions between the particles [46] and can be modelled as presented by Jolly et al. [50].

As can be seen in Figure 10, the viscous flow mechanism appears to be the main contribution to damping as the average tan *δ* of neat PDMS matrix was ~0.155. The inclusion of the particles enabled additional damping mechanisms, however, the tan *δ* of the MRE containing bare CI was only ~0.138 due to limited bonding of the CI particles that rather represented micro-cavities in the body of the matrix (Figure 6) therefore the damping mechanism for strongly bonded interfaces is not relevant. On the other hand, the interfacial damping (for weakly bonded particles) and magnetism-induced damping were manifested in the MRE containing CI-*g*-PHEMATMS-1 particles very well, which resulted in total damping factor of ~0.208. Further enhancement in damping factor was achieved by the incorporation of CI-*g*-PHEMATMS-2 particles and the MRE containing these particles exhibited the average damping factor of ~0.234. Assuming the same magnetism-induced damping in both modified systems, it can be concluded that the enhancement in the latter system (CI-*g*-PHEMATMS-2) was mainly caused by additional interfacial friction and thus higher energy dissipation as a result of higher-molecular weight PHEMATMS grafts occurrence.

### 3.6. Field-Induced Changes in Surface Topography

The bulk properties of the MREs were the subject of numerous studies [1,4,10,13,18,20,21,26,29,40,44,51,52,53,54] however, the research of their surface properties is still limited. Yet, the MREs were recently shown to be exceptional materials with tunable surface roughness [55], which opens new avenues in the context of possible applications. The MREs based on matrices with high stiffness do not exhibit any surface-responsivity as the particle restructuration is not manifested in changes on the free surface [56]. On the other hand, the MREs based on liquid-state matrices such as uncured PDMS show super-hydrophobicity and substantial changes in surface properties, but the employment of the liquid-state matrix can be a limiting factor in certain applications [57]. Recently, Glavan et al. [55] developed the MRE film based on cross-linked PDMS and the suitable matrix stiffness ensured by the incorporation of silicone oil as a plasticizing agent. The matrix stiffness thus plays a key role in achieving the MREs with tunable surface properties. As we have shown above (Figure 6), the MREs’ stiffness can be modulated via the ATRP particle-grafting technique, which was presumed to affect also the surface properties. Therefore, the next part of this study is focused on the investigation of topography changes of the MREs containing bare CI particles or their PHEMATMS-grafted analogues. 

**Figure 11:**
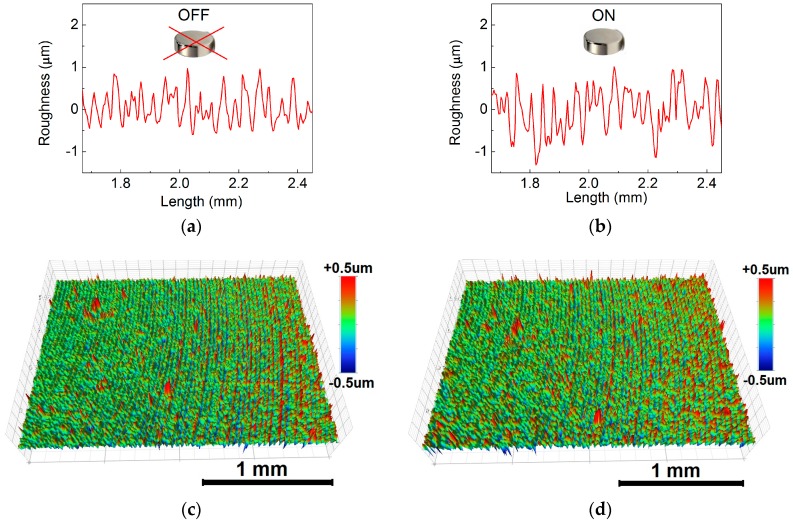
Representative (**a**,**b**) 2D height profiles and (**c**,**d**) 3D surface topography images of the MRE sheet containing bare CI particles at (**left**) the off-state and (**right**) in the presence of the external magnetic field.

**Figure 12:**
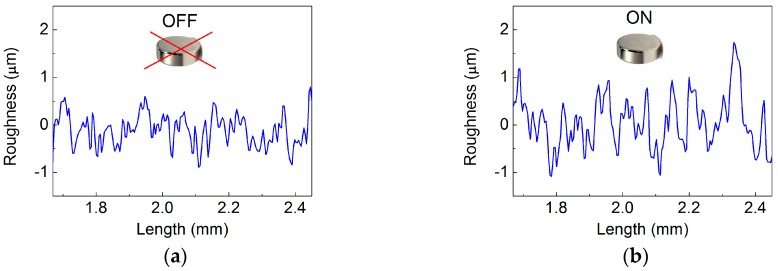

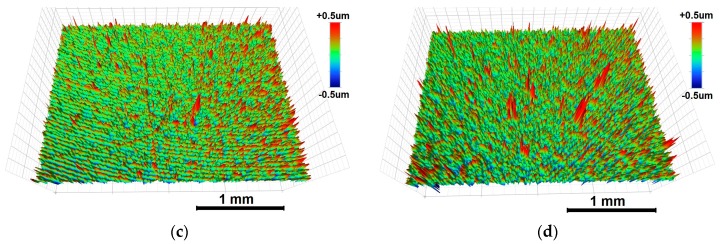
Representative (**a**,**b**) 2D height profiles and (**c**,**d**) 3D surface topography images of the MRE sheet containing CI-*g*-PHEMAMTS-1 particles at (**left**) the off-state and (**right**) in the presence of the external magnetic field.

The relevant parameters qualitatively describing the effect of the external magnetic field on changes of the MRE sheet surfaces are outlined in Table 3. The changes were first evaluated using the average maximum height of the surface, ${\bar{R}}_{Z}$. This quantity takes into account the average values of the 10 highest peaks, and the ten lowest valleys found over the complete 3D image giving the idea about the overall peak to valley magnitude of the surface. As can be seen, all the investigated MREs were characterized by similar ${\bar{R}}_{Z}$ values in the absence of magnetic field as the same mold was used during their fabrication (the same initial roughness ensured). However, after the magnetic field application the MRE containing bare CI particles exhibited only small ~14%, relative change in the ${\bar{R}}_{Z}$ values when compared with its analogues containing the PHEMATMS-grafted particles that achieved changes in ${\bar{R}}_{Z}$ values of ~49% and ~67%, respectively. The results correlate with the *G′* of the MREs (Figure 6) showing that larger relative motion of the particles can be attained in the composites with lower *G′*, which is manifested in more pronounced topographical restructuring. To further quantify the changes in the MRE surface roughness the arithmetical mean height of a line extended to the analyzed surface, ${\bar{S}}_{A}$, was analyzed. This parameter expresses, as an absolute value, the difference in height of each point compared to the arithmetical mean of the surface and is generally used to evaluate surface roughness. The average ${\bar{S}}_{A}$ values from five measurements on each sample are included in Table 3. The representative 2D height profiles for the MRE sheet containing bare CI particles are displayed in Figure 11a,b, while detailed 3D surface topographies are shown in Figure 11c,d. The data for the MRE containing the CI-*g*-PHEMATMS-1 particles are presented analogously in Figure 12a–d. As can be seen, the quality of the surface was in all the cases balanced without any distinct imperfections. In the presence of magnetic field, the particles have a tendency to be preferentially oriented along the magnetic flux lines [55]. Comparing the 3D images, one can notice that any distinct surface changes were apparent in the reference sample, while the surface roughness was notably enhanced in the case of modified MRE. The results for the MRE containing the CI-*g*-PHEMATMS-2 are presented numerically (Table 3). The restructuration process was predominant in the MREs with suitably-soft matrices, which in our case was ensured by the incorporation of PHEMATMS-grafted particles. The ability of inducing the “conical”, or the “mountain” structures [55] that are uniformly distributed within the sample can find application as wettability-modulating or self-cleaning surfaces [58].

## 4. Conclusions

In this paper, two types of CI-*g*-PHEMATMS particles varying in molecular weight (${\bar{M}}_{n}$ of ~9200, and ~17,900 g·mol^−1^) of their grafts were synthesized via SI-ATRP, and the multiple-function of PHEMATMS grafts on the performance of their PDMS-based isotropic MREs of 60 wt % concentration was investigated. The compact PHEMATMS layers enhanced stability of the CI particles and prevented their degradation in strong acidic environment (pH of 1). The presence of PHEMATMS further improved bulk properties of the MREs by modifying the particles/matrix interface. Despite lowering the *G′*, the presence of CI-*g*-PHEMATMS particles probably loosened the PDMS cross-link density which facilitated the particle rearrangement in magnetic fields. Although modified particles exhibited slightly lower magnetization, their MREs were able to develop higher relative MR effects (enhancing factor of 1.54, and 1.78 above the reference) when compared to bare CI particles, and their MREs, respectively. The modified particle/matrix interface was also more effective in damping due to higher energy dissipation as a result of PHEMATMS grafts occurrence, which was the most significant in the MRE containing the particles grafted with PHEMATMS of higher molecular weight. The plasticizing effect of PHEMATMS grafts also facilitated the field-induced topological restructuring of the MRE sheets resulting in notable surface roughness changes (Δ${\bar{S}}_{A}$ of ~37, and ~63%) when compared with the reference (Δ${\bar{S}}_{A}$ of ~13%) containing bare CI particles. The ATRP particle-grafting technology is a versatile technique in the technology of the MREs that enabled tuning the MR performance, damping capacity with a considerable impact on surface roughness of the MREs, while significantly enhancing stability properties of the magnetic particles with a negligible impact on their magnetization.

## Figures and Tables

**Table 1:** Reaction stoichiometry and experimental conditions for SI-ATRP of PHEMATMS grafts.

Sample Code	M ^1^	I ^1^	CuBr	L ^1^	Anisole (vol.%)	Time (h)
CI-*g*-PHEMATMS-1	100	1	1	1	50	2
CI-*g*-PHEMATMS-2	300	1	1	1	50	2

^1^ M, I, and L denote monomer (HEMATMS), macroinitiator (EBiB), and ligand (PMDETA), respectively.

**Table 2:** Results of CI-*g*-PHEMATMS particle syntheses.

Sample Code	Conversion ^1^ (%)	${\bar{M}}_{W}$ (g·mol^−1^)	${\bar{M}}_{n}$ (g·mol^−1^)	*Đ* (−)
CI-*g*-PHEMATMS-1	90	11,800	9200	1.28
CI-*g*-PHEMATMS-2	75	23,500	17,900	1.31

^1^ based on ^1^H NMR spectra.

**Table 3:** Parameters describing the topography of the MRE sheets.

MRE filler	Bare CI		CI-*g*-PHEMATMS-1		CI-*g*-PHEMATMS-2	
Magnet	OFF	ON	OFF	ON	OFF	ON
${\bar{R}}_{Z}$ (μm)	10.1	11.5	9.98	14.9	10.6	17.7
Δ${\bar{R}}_{Z}$ (%)	~14		~49		~67	
${\bar{S}}_{A}$ (μm)	0.39	0.44	0.41	0.56	0.43	0.70
Δ${\bar{S}}_{A}$ (%)	~13		~37		~63	
